# Altered neuronal habituation to hearing others’ pain in adults with autistic traits

**DOI:** 10.1038/s41598-020-72217-x

**Published:** 2020-09-14

**Authors:** Jing Meng, Zuoshan Li, Lin Shen

**Affiliations:** 1 Key Laboratory of Applied Psychology, Chongqing Normal University, Chongqing, China; 2 School of Education, Chongqing Normal University, Chongqing, China; 3 School of Mathematical Sciences, Chongqing Normal University, Chongqing, China

**Keywords:** Cognitive neuroscience, Emotion, Sensory processing, Human behaviour

## Abstract

This study tested the hypothesis that autistic traits influence the neuronal habituation that underlies the processing of others’ pain. Based on their autism-spectrum quotient (AQ), two groups of participants were classified according to their autistic traits: High-AQ and Low-AQ groups. Their event-related potentials in response to trains of three identical audio recordings, exhibiting either painful or neutral feelings of others, were compared during three experimental tasks. (1) In a Pain Judgment Task, participants were instructed to focus on pain-related cues in the presented audio recordings. (2) In a Gender Judgment Task, participants were instructed to focus on non-pain-related cues in the presented audio recordings. (3) In a Passive Listening Task, participants were instructed to passively listen. In the High-AQ group, an altered empathic pattern of habituation, indexed by frontal-central P2 responses of the second repeated painful audio recordings, was found during the Passive Listening Task. Nevertheless, both High-AQ and Low-AQ groups exhibited similar patterns of habituation to hearing others’ voices, both neutral and painful, in the Pain Judgment and Gender Judgment Tasks. These results suggest altered empathic neuronal habituation in the passive processing of others’ vocal pain by individuals with autistic traits.

## Introduction

Empathy is the ability of an individual to comprehend other peoples’ thoughts and feelings as if these thoughts and feelings were experienced by the perceiving individual^1,2^. Empathy plays an important role in social interactions and daily lives^3^. By extension, empathy for pain implies the ability of an individual to understand and evaluate the suffering of others^4^. This enables typically developing individuals to understand the feelings of another individual who is in pain and improve the regulation of appropriate social behaviour^5^. The perception of others’ pain may automatically elicit corresponding representations in the perceiving individual, so that one could involuntarily match the suffering of others. In addition, if expressed visually^6,7^ or vocally^8^, empathy for others’ pain depends on the level of attention to the stimulus.

Autism spectrum disorder (ASD) is characterised by persistent impairments in social interactions, and by the presentation of restricted and repetitive patterns of behaviour, interests, or activities^9^. Previous studies have indicated that the quantifiable autistic traits included in the ASD core deficits are continuously distributed in typically developing individuals^10^. The autism-spectrum quotient (AQ)^11^ has been used to estimate autistic traits in both ASD individuals and typically developing individuals. ASD individuals were usually identified by extremely high AQ scores compared with the general population^12^. Empathic impairments in the response to others’ feelings have been suggested as a key symptom of ASD^13^. Previous studies suggested that typically developing individuals with high AQ scores (i.e., individuals with autistic traits or High-AQ individuals) exhibit similar behavioural patterns of their empathic responses compared with individuals with ASD^14,15^. Compared with individuals with low AQ scores (i.e., Low-AQ individuals), High-AQ individuals exhibit impaired empathy in implicit tasks, i.e., when their attention is directed away from others’ emotional cues^16,17^.

Habituation is a fundamental property of cortical neural responses and has been suggested to be altered in individuals with ASD^18^. Habituation is pervasive in sensory systems; it refers to changes in neural and behavioural responses that accompany prolonged exposure to an adapting stimulus with repeated features^19^. Altered habituation in individuals with ASD and autistic traits has been found across diverse domains, including tactile stimulation^20^, face discrimination^21^, audio-visual asynchrony^22^, saccade amplitude^23^, and auditory cortical adaptation^18,19,24–26^. For example, a fMRI study used pure tones, which were repeatedly presented at a short and constant inter-stimulus interval (ISI) to individuals with ASD to characterize neural adaption across time. The study found that the post-transient sustained response to the fixed-interval timing repeated tone was stronger in ASD participants than in controls; this reflects reduced auditory adaptation in individuals with ASD^18^. Previous studies typically used pure tones to study auditory habituation in individuals with ASD and autistic traits. However, whether individuals with autistic traits would exhibit impaired empathic adaptation to repeated emotional auditory stimuli remains an open question.

An association between individuals with ASD or autistic traits and their selective impairment with regard to judging others’ pain in the visual^27^ and auditory^17,28^ modalities has been documented. In addition, the effects of the modulation of top-down attention on the empathic abilities of individuals with autistic traits have also been studied. In line with previous studies^7,8^, our previously published study explored top-down attention for others’ pain, which was manipulated by instructing participants to either focus on pain cues in the stimuli, or to focus on non-pain cues^16,17^. The results showed that if individuals with autistic traits were not explicitly instructed to focus on others’ feelings, their empathic responses show impairments^16,17^. Furthermore, previous studies indicated that during passive viewing, ASD individuals did not show empathic responses until they were explicitly instructed to do so; this suggests that in these individuals, the spontaneous empathic response may be impaired^29,30^. Those studies presented important aspects for the application of experimental paradigms in High-AQ participants. Differences in the top-down attention of task instructions can lead to contrasting empathic responses by these participants.

The present study attempted to explore whether empathic habituation to others’ pain in individuals with autistic traits is modulated by top-down attention in the auditory modality with event-related potentials (ERPs). Consistent with our previous study^8^, participants received different instructions for three tasks: (1) Pain Judgment Task: participants were asked to determine the pain level of the presenters of audio recordings; participants had to focus on pain-related cues in this task. (2) Gender Judgment Task: participants were asked to judge the gender of the presenters of audio recordings; participants had to focus on non-pain cues in this task. (3) Passive Listening Task: participants were asked to listen passively without receiving any explicit instruction. This task minimises the above-mentioned influences of instructions, and represents individuals’ spontaneous behaviour and neural responses under natural conditions.

Two hypotheses were tested in the present study. First, previous studies indicated that ASD individuals did not show empathic responses without specific instruction to pay attention to others^29,30^. Based on this, the present study hypothesized that altered empathic neuronal habituation to others’ painful voices in the High-AQ group would only be found in the Passive Listening Task. This represents the impaired empathic habituation of individuals with autistic traits to others’ pain, when they have not been specifically instructed to focus their attention to others (regardless of the presence of pain or non-pain cues). Second, previous studies indicated that the empathic responses of individuals with autistic traits showed impairments in implicit tasks^16,17^. Based on this, the present study hypothesized that altered empathic neuronal habituation in the High-AQ group will be present in both the Passive Listening Task and the Gender Judgment Task. In addition, since a previous study found a specific relationship between the magnitude of autistic traits and empathic behavioural responses^14^, the present study predicted that individuals’ degree of self-reported AQ scores will be associated with their empathic neuronal habituation. Accordingly, individuals with autistic traits show a selective deficit in the empathic neuronal habituation to hearing others’ painful voices.

## Results

**Figure 1:**
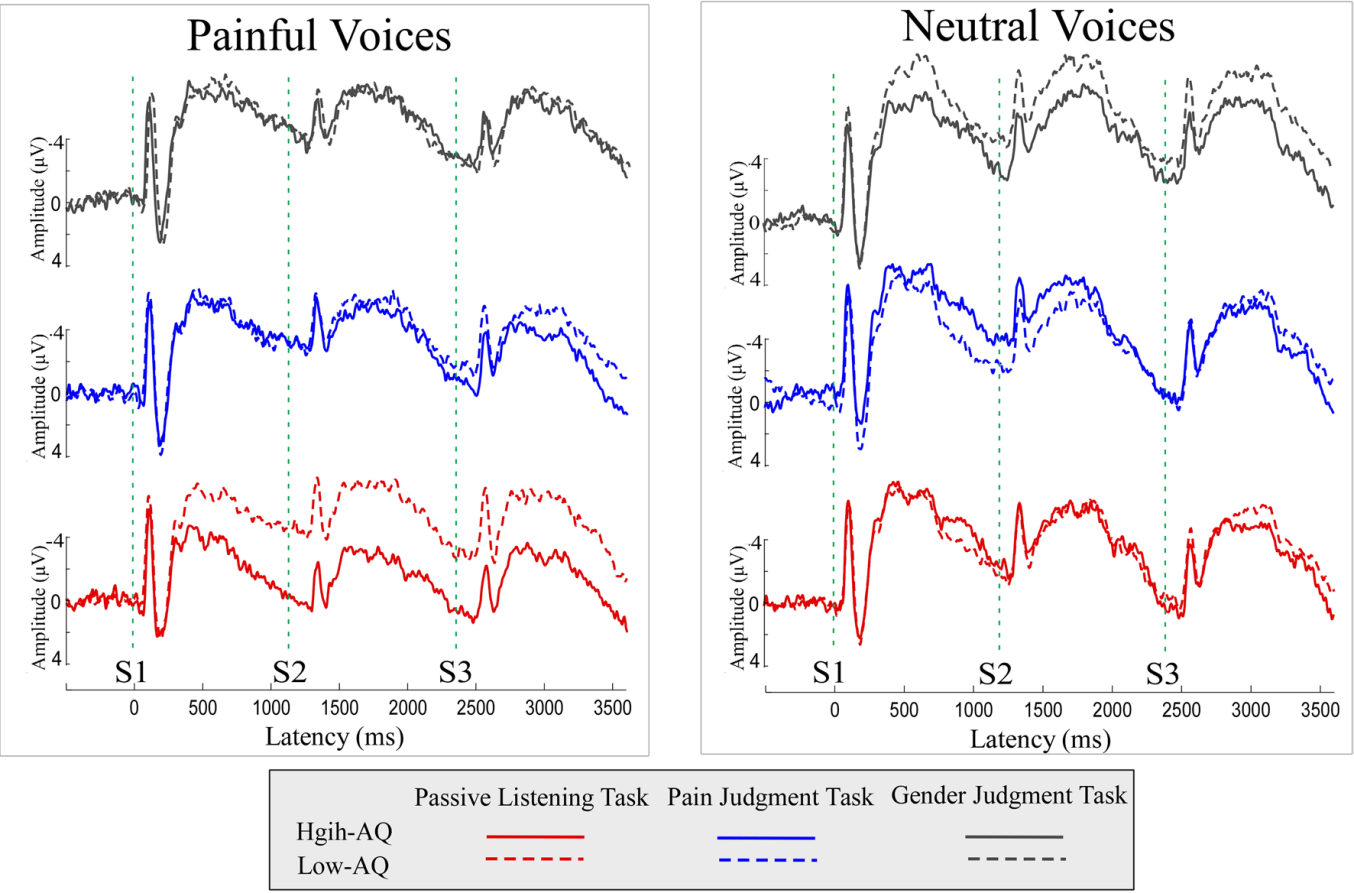
Neuronal responses to hearing others’ painful and neutral voices. Event-related potential waveforms were elicited by hearing others’ painful (left column) and neutral (right column) voices during the Pain Judgment Task (blue lines), Gender Judgment Task (grey lines), and Passive Listening Task (red lines). The respective recordings were played for participants in High-AQ (solid lines) and Low-AQ (dotted lines) groups. The displayed signals were measured from the frontal-central electrodes (Fz, F1, F2, FCz, FC1, and FC2). The onset times of S1, S2, and S3 are illustrated with green straight dotted lines.

**Figure 2:**
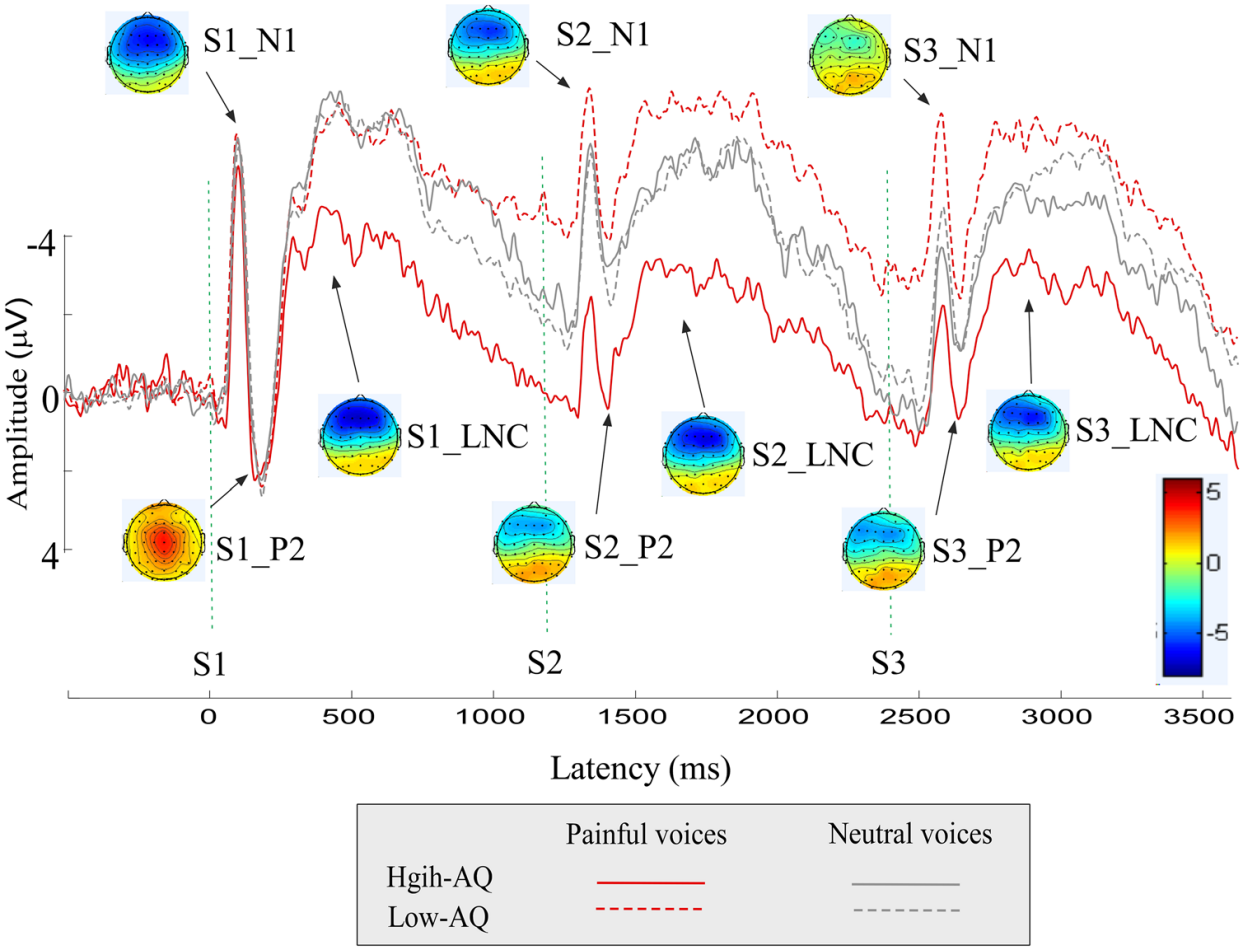
Temporal and spatial characteristics of the neuronal responses to hearing others’ painful and neutral voices during the Passive Listening Task. Event-related potential waveforms were elicited by hearing others’ painful (red lines) and neutral (grey lines) voices during the Passive Listening Task. The respective recordings were played for High-AQ (solid lines) and Low-AQ (dotted lines) groups. Scalp topographies of the dominant ERP components (N1, P2, and LNC) were computed at respective peak latencies.

No significant differences in the accuracies (ACCs) and reaction times (RTs) were identified in the Pain Judgment and Gender Judgment Tasks between the High-AQ and Low-AQ groups. Detailed statistical comparisons of behavioural data are summarized in the Supplementary Materials (S. Table 1). Grand average ERP waveforms to hearing others’ painful and neutral voices as well as scalp topographies of dominant waves are shown in Figs. 1 and 2. Consistent with previous studies, hearing others’ painful voices evoked more negative N1 and late negative complex (LNC) components, with maximal distributions over frontal-central electrodes, regardless of the repetition of vocal stimulation. The first audio recording (S1) in the triplet evoked a centrally distributed P2 response, which was less dominant during the second and third audio recordings (S2 and S3).

### Frontal-central N1 component

As shown in Figs. 1 and 2 and S. Figure 1 in the Supplementary Materials, the frontal-central S1_N1 amplitude was significantly modulated by the interactions of “group”, “task”, and “stimulation” (*F*_*2, 37*_ = 4.71, *p* = 0.015, η_p_^2^ = 0.20). Simple effects analyses indicated that, for High-AQ groups, the effect of “task” was found in response to neutral voices (*F*_*2, 37*_ = 3.48, *p* = 0.041, η_p_^2^ = 0.16). However, this effect did not pass FDR correction. Also, S1_N1 amplitudes were not different among experimental tasks in other conditions (*p* > 0.05 for all comparisons).

**Figure 3:**
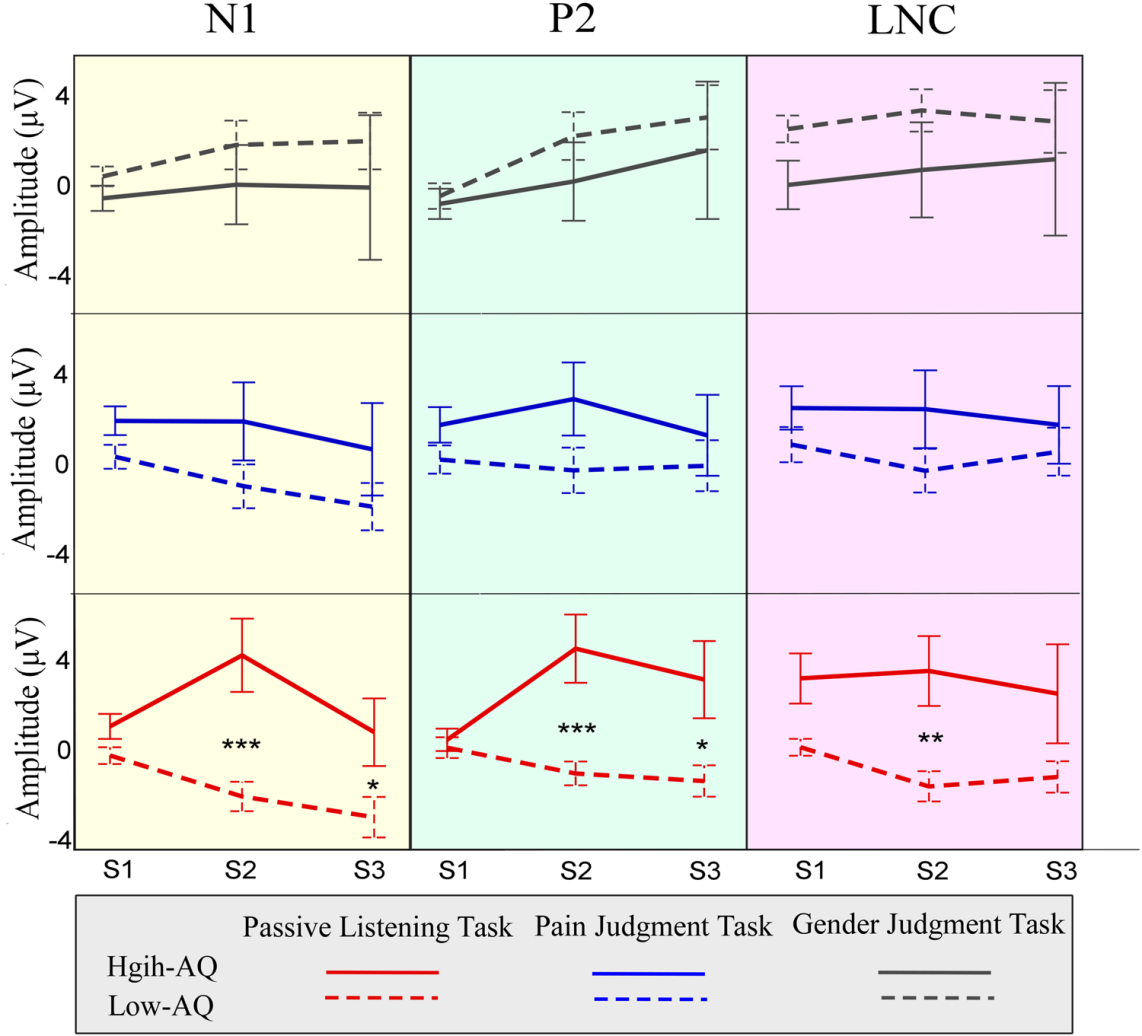
Differential ERP amplitudes between painful and neutral voices. Differential ERP amplitudes between painful and neutral voices (painful-neutral) of N1 (left panel), P2 (middle panel), and LNC (right panel) components, elicited by the onset of S1, S2, and S3 audio recordings, were compared between High-AQ (solid lines) and Low-AQ (dotted lines) groups during the Pain Judgment Task (blue lines), Gender Judgment Task (grey lines), and Passive Listening Task (red lines). The High-AQ group exhibited altered neuronal habituation to repeated painful voices during the Passive Listening Task. Data in the line charts are expressed as Mean ± SEM. *: *p* < 0.05, **: *p* < 0.01, ***: *p* < 0.001.

The frontal-central S2_N1 amplitude was significantly modulated by the main effect of “task” (*F*_*2, 37*_ = 5.02, *p* = 0.012, η_p_^2^ = 0.21). Post hoc comparisons showed that S2_N1 amplitudes in the Passive Listening Task were significantly higher than in the Gender Judgment Task (− 4.06 ± 0.57 μV vs. − 6.37 ± 0.45 μV, *p* = 0.003). However, no significant differences were observed between the Pain Judgment Task and the other two tasks (*p* > 0.05 for all comparisons). The S2_N1 amplitude was significantly modulated by the interactions of “group × task” (*F*_*2, 37*_ = 4.50, *p* = 0.018, η_p_^2^ = 0.20), “group × stimulation” (*F*_*2, 37*_ = 4.87, *p* = 0.033, η_p_^2^ = 0.11), and “group × task × stimulation” (*F*_*2, 37*_ = 4.14, *p* = 0.024, η_p_^2^ = 0.18). Simple effects analyses indicated that, with regard to painful voices in the Passive Listening Task, S2_N1 amplitudes were significantly higher for Low-AQ groups than for High-AQ groups (− 6.71 ± 4.34 μV vs. − 0.43 ± 5.99 μV, *F*_*2, 37*_ = 14.45, *p* = 0.001, η_p_^2^ = 0.275), which passes FDR correction. S2_N1 amplitudes were not different between groups in other conditions (*p* > 0.05 for all comparisons). In addition, the group differences of differential ERP amplitudes between painful and neutral voices (i.e., painful-neutral) were significant in the Passive Listening Task (*p* = 0.001), but neither in the Pain Judgment Task (*p* = 0.160), nor the Gender Judgment Task (*p* = 0.392) (see Fig. 3).

The frontal-central S3_N1 amplitude was only significantly modulated by the main effect of “task” (*F*_*2, 37*_ = 5.60, *p* = 0.007, η_p_^2^ = 0.23). Post hoc comparisons showed that S3_N1 amplitudes in the Gender Judgment Task (−3.48 ± 0.75 μV) were significantly higher than those in both the Passive Listening Task (−0.62 ± 0.71 μV, *p* = 0.006) and the Pain Judgment Task (− 0.86 ± 0.73 μV, *p* = 0.008). No significant differences were observed between the Pain Judgment Task and the Passive Listening Task (*p* = 0.821).

### Central P2 component

The S1_P2 amplitude was only significantly modulated by the main effect of “task” (*F*_*2,37*_ = 3.41, *p* = 0.044, η_p_^2^ = 0.16). Post hoc comparisons showed that the S1_P2 amplitudes in the Passive Listening Task (2.71 ± 0.42 μV) were significantly smaller than those in the Pain Judgment Task (3.67 ± 0.54 μV, *p* = 0.004). In addition, S1_P2 amplitudes in response to painful voices in the Passive Listening Task (2.68 ± 3.02 μV) were smaller than those in the Pain Judgment Task (3.99 ± 3.51 μV) (*t* = − 3.24, *p* = 0.002) (for more details, see S. Table 2, Supplementary Materials). No other significant differences were observed between tasks (*p* > 0.05 for all comparisons).

The S2_P2 amplitude was significantly modulated by the main effects of “task” (*F*_*2, 37*_ = 5.02, *p* = 0.012, η_p_^2^ = 0.21) and “stimulation” (*F*_*2, 37*_ = 5.02, *p* = 0.012, η_p_^2^ = 0.21). Post hoc comparisons showed that the S2_P2 waves in the Gender Judgment Task (− 3.24 ± 0.64 μV) were more negative than those in both the Passive Listening Task (− 1.56 ± 0.61 μV, *p* = 0.021) and the Pain Judgment Task (− 2.14 ± 0.61 μV, *p* = 0.042). However, no significant differences were observed between the Pain Judgment Task and the Passive Listening Task (*p* = 0.370). Painful voices elicited smaller S2_P2 amplitudes than neutral voices (− 2.92 ± 0.60 μV vs. − 1.71 ± 0.54 μV). The S2_P2 amplitude was significantly modulated by the interaction of “group × task × stimulation” (*F*_*2, 37*_ = 4.39, *p* = 0.016, η_p_^2^ = 0.10). Simple effects analyses indicated that, when reacting to painful voices in the Passive Listening Task, S2_P2 amplitudes were significantly smaller for Low-AQ groups than for High-AQ groups (− 3.21 ± 3.55 μV vs. 1.46 ± 6.20 μV, *F*_*2, 37*_ = 8.54, *p* = 0.006, η_p_^2^ = 0.18), which passed FDR correction. The S2_P2 amplitudes were not different between groups in other conditions (*p* > 0.05 for all comparisons). In addition, group differences of differential ERP amplitudes between painful and neutral voices (i.e., painful-neutral) were significant in the Passive Listening Task (*p* = 0.001), but neither in the Pain Judgment Task (*p* = 0.108), nor the Gender Judgment Task (*p* = 0.324) (see Fig. 3).

The S3_P2 amplitude was only significantly modulated by the main effect of “task” (*F*_*2, 37*_ = 3.42, *p* = 0.043, η_p_^2^ = 0.16). Post hoc comparisons showed that the S3_P2 amplitudes in the Gender Judgment Task (-4.10 ± 0.78 μV) were significantly smaller than those in the Pain Judgment Task (− 2.00 ± 0.78 μV, *p* = 0.023). No significant differences were observed between the Passive Listening Task (− 2.33 ± 0.76 μV) and the other two tasks (*p* > 0.05 for all comparisons).

### Frontal-central LNC component

The S1_LNC and S2_LNC amplitudes were significantly modulated by the main effects of “task” (S1_LNC: *F*_*2, 37*_ = 5.14, *p* = 0.011, η_p_^2^ = 0.22; S2_LNC: *F*_*2, 37*_ = 4.70, *p* = 0.015, η_p_^2^ = 0.20) and “stimulation” (S1_LNC: *F*_*2, 37*_ = 21.94, *p* < 0.001, η_p_^2^ = 0.37; S2_LNC: *F*_*2, 37*_ = 4.48, *p* = 0.041, η_p_^2^ = 0.11). Post hoc comparisons showed that both the S1_LNC and S2_LNC waves in the Gender Judgment Task (S1_LNC: − 7.27 ± 0.74 μV; S2_LNC: − 7.63 ± 0.77 μV) were more negative than those in both the Passive Listening Task (S1_LNC: − 5.87 ± 0.61 μV, *p* = 0.004; S2_LNC: − 5.23 ± 0.72 μV, *p* = 0.009) and the Pain Judgment Task (S1_LNC: − 6.30 ± 0.63 μV, *p* = 0.013; S2_LNC: − 5.82 ± 0.74 μV, *p* = 0.014). No significant differences were observed between the Pain Judgment Task and the Passive Listening Task (S1_LNC: *p* = 0.253; S2_LNC: *p* = 0.474). Painful voices elicited more positive waves than neutral voices (S1_LNC: − 5.75 ± 0.64 μV vs. − 7.22 ± 0.64 μV; S2_LNC: − 5.59 ± 0.64 μV vs. − 6.87 ± 0.67 μV). S1_LNC and S2_LNC amplitudes were modulated by the interaction of “group × task × stimulation” (S1_LNC: *F*_*2, 37*_ = 4.69, *p* = 0.015, η_p_^2^ = 0.20; S2_LNC: *F*_*2, 37*_ = 3.93, *p* = 0.028, η_p_^2^ = 0.18). Simple effects analyses indicated that, when reacting to painful voices in the Passive Listening Task, both the S1_LNC and S2_LNC amplitudes were significantly larger for Low-AQ groups than for High-AQ groups [S1_LNC: − 6.46 ± 3.94 μV vs. − 3.60 ± 4.89 μV, *F*_*2, 37*_ = 4.14, *p* = 0.049, η_p_^2^ = 0.10 (did not pass FDR correction); S2_LNC: − 7.32 ± 4.42 μV vs. − 2.16 ± 6.32 μV, *F*_*2, 37*_ = 8.96, *p* = 0.005, η_p_^2^ = 0.19 (passed FDR correction)]. The S1_LNC and S2_LNC amplitudes were not different between groups in the other conditions (*p* > 0.05 for all comparisons). In addition, for the S2_LNC component, the group differences of the differential ERP amplitudes between painful and neutral voices (i.e., painful-neutral) were significant in the Passive Listening Task (*p* = 0.005), but neither in the Pain Judgment Task (*p* = 0.179), nor the Gender Judgment Task (*p* = 0.256) (see Fig. 3).

The frontal-central S3_LNC amplitude was only significantly modulated by the main effect of “task” (*F*_*2, 37*_ = 3.42, *p* = 0.043, η_p_^2^ = 0.16). Post hoc comparisons showed that the S3_LNC amplitudes in the Gender Judgment Task (− 7.23 ± 1.01 μV) were significantly larger than those in both the Pain Judgment Task (− 4.80 ± 0.95 μV, *p* = 0.036) and the Passive Listening Task (− 4.72 ± 0.84 μV, *p* = 0.028). No significant differences were observed between the Pain Judgment Task and the Passive Listening Task (*p* = 0.840).

### Indexes of empathic neuronal habituation

**Figure 4:**
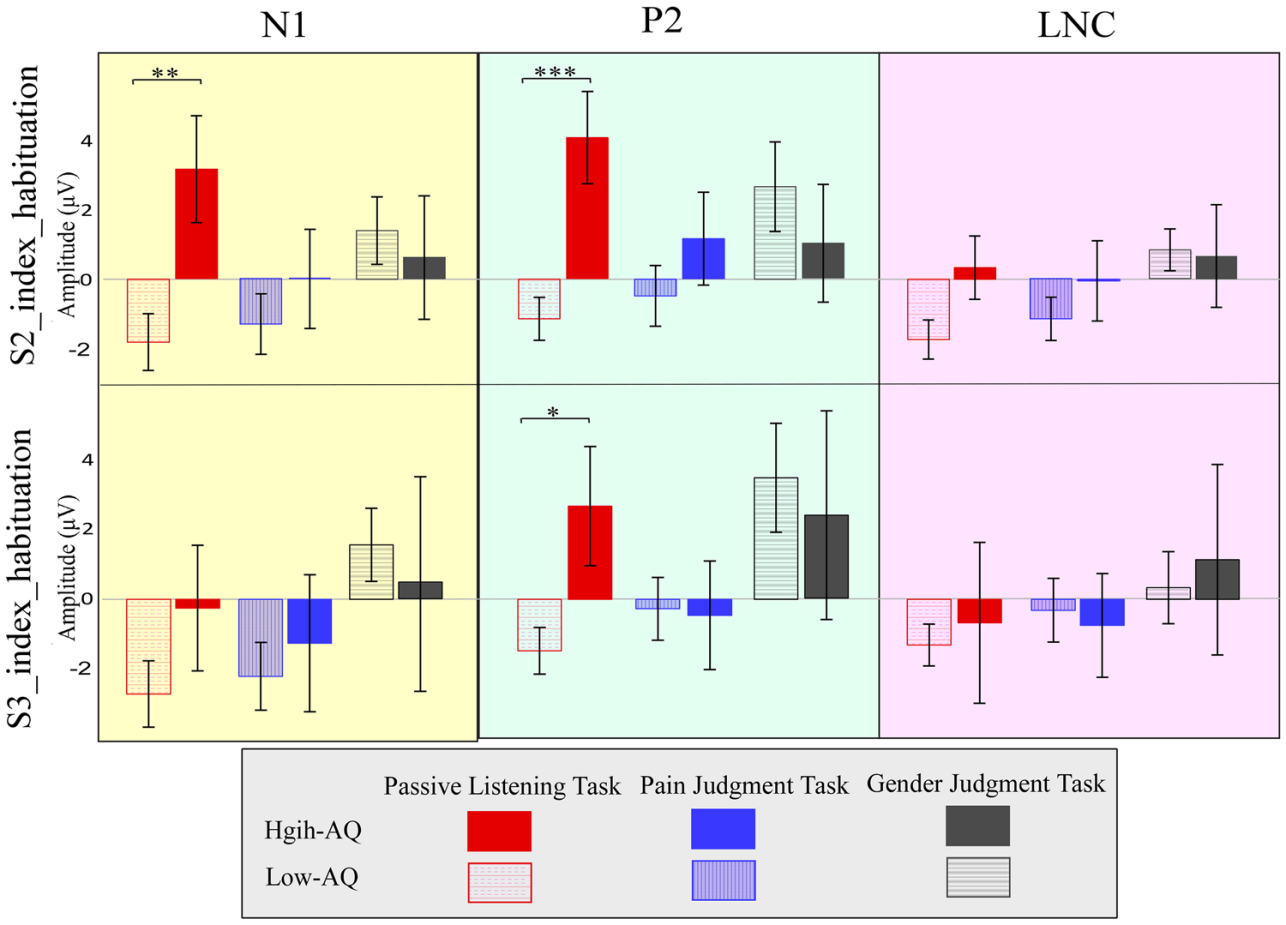
Comparisons of amplitudes of the indexes for neuronal habituation between groups. The S2_Index_habituation (top panel) and S3_Index_habituation (bottom panel) of N1 (left panel), P2 (middle panel), and LNC (right panel) amplitudes were compared between High-AQ (solid bars) and Low-AQ (dotted bars) groups during the Pain Judgment Task (blue bars), Gender Judgment Task (grey bars), and Passive Listening Task (red bars). The High-AQ group exhibited altered neuronal habituation of empathic responses to others’ vocal pain during the Passive Listening Task. Data in bar charts are expressed as Mean ± SEM. *: *p* < 0.05, **: *p* < 0.01, ***: *p* < 0.001.

Detailed statistical comparisons of the indexes of empathic neuronal habituation are shown in Table 1 and Fig. 4.

**Table 1:** Summary of statistical analyses of indexes of empathic neuronal habituation.

	Task			Group			Task × Group		
	*F*_*2,37*_	*p*	η_p_^2^	*F*_*1,38*_	*p*	η_p_^2^	*F*_*2,37*_	*p*	η_p_^2^
**S2_index_habituation**									
S2_N1_habituation	0.93	0.405	0.05	2.89	0.097	0.07	2.56	0.091	0.12
S2_P2_habituation	0.83	0.446	0.04	2.82	0.101	0.07	**3.53**	**0.040**	**0.16**
S2_LPC_habituation	1.95	0.157	0.10	1.04	0.314	0.03	1.08	0.349	0.06
**S3_index_habituation**									
S2_N1_habituation	1.78	0.182	0.09	0.16	0.689	0.004	0.67	0.520	0.04
S2_P2_habituation	2.73	0.080	0.07	0.28	0.598	0.01	1.87	0.167	0.05
S2_LPC_habituation	0.86	0.432	0.04	0.04	0.851	0.001	0.10	0.908	0.01

Results were obtained using repeated measures ANOVA with the within-participant of “task” and the between-participant of “group”. Significant (*p* < 0.05) comparisons are indicated in boldface.

The amplitudes of S2_P2_habituation were significantly modulated by the “group × task” interaction (*F*_*2, 37*_ = 3.53, *p* = 0.040, η_p_^2^ = 0.16). Simple effects analyses indicated that, with regard to the differences between painful and neutral voices in the Passive Listening Task, the amplitudes of S2_P2_habituation were significantly larger for High-AQ groups than for Low-AQ groups (4.01 ± 5.88 μV vs. − 1.13 ± 2.74 μV; *F*_*1, 38*_ = 12.60, *p* = 0.001, η_p_^2^ = 0.25), which passed FDR correction. In the Pain Judgement Task, the amplitudes of S2_P2_habituation were not different between groups (*F*_*1, 38*_ = 1.04, *p* = 0.314, η_p_^2^ = 0.03) and the same was found for the Gender Judgement Task (*F*_*1, 38*_ = 0.61, *p* = 0.439, η_p_^2^ = 0.02). No other main effect or interaction was found (*p* > 0.05 for all comparisons).

To investigate whether the indexes of empathic neuronal habituation correlated with AQ scores, the Pearson Correlation between them was calculated. Amplitudes of S2_N1_habituation (*r*_*40*_ = 0.34, *p* = 0.035) and S2_P2_habituation (*r*_*40*_ = 0.42, *p* = 0.007) in the Passive Listening Task were significantly correlated with AQ scores. No other reliable correlation was found between AQ scores and indexes of empathic neuronal habituation (*p* > 0.05 for all correlations).

## Discussion

The present study investigated the influence of autistic traits on the top-down attention-induced modulation of the habituation to others’ vocalizations of pain. In line with previous results^8^, top-down attention to another individual’s pain was manipulated by instructing participants to either focus on pain-related cues, non-pain cues, or by passively listening to the provided audio recordings. ERPs were used to measure the empathic neural responses that were induced by the repetition of three identical audio recordings (S1–S2–S3) with short and constant ISI in both High-AQ and Low-AQ groups. The indexes for empathic neuronal habituation were analysed in each condition. The results showed that the ERP responses to the first stimulus (S1) were equivalent between groups. However, in the Passive Listening Task, the ERP amplitudes in response to painful voices in the second (S2) and third (S3) stimuli of the triplet (S2_N1, S2_P2, S2_LNC, S3_N1, and S3_P2) were lower in the High-AQ group than in the Low-AQ group. More importantly, in the Passive Listening Task, the amplitudes of S2_P2_habituation were only significantly higher for High-AQ groups rather than for Low-AQ groups. This reflects the altered habituation of neuronal empathic responses to others’ vocal pain, when individuals with autistic traits have not been instructed to specifically focus their attention to others.

Consistent with the results of a previous study^8^, in the present study, the P2 amplitudes of S1 in response to the painful voices in the Passive Listening Task were smaller than in the Pain Judgment Task. Since the P2 response is relevant to the emotional quality of the audio recordings^31^, and since our previous studies showed that the P2 amplitudes related to painful voices were significantly and positively correlated with pain intensity ratings^8,17^, P2 amplitudes to painful voices at least partly reflected cognitive empathy for others’ vocal pain^8^. This suggests that, compared with the Passive Listening Task, when focusing on others’ vocal pain in the Pain Judgement Task, participants showed more empathic neuronal responses to others’ pain. Consequently, top-down attention could modulate individuals’ empathic responses to others’ vocal pain.

Consistent with previous studies^26,32–35^, the present study showed that stimulus repetition at short and constant ISI significantly decreased the magnitudes of S2 and S3 stimuli (see Figs. 1 and 2). This observation can be interpreted as a consequence of auditory habituation. Habituation is a neural regulatory process that adjusts neural responses to the current sensory environment in response to repeated presentation of stimuli, and often involves response reductions^36^. The ERP results of the present study indicate that, for most conditions (i.e., painful and neutral voices in the Pain Judgment Task and Gender Judgment Task, as well as neutral voices in the Passive Listening Task), both groups exhibited comparable auditory habituation.

Interestingly, analyses of group differences only found significant differences in the indexes of empathic neuronal habituation to others’ vocal pain between High-AQ and Low-AQ groups in the S2_P2_habituation of the Passive Listening Task. In addition, in accordance with a previous study^14^, amplitudes of S2_P2_habituation in the Passive Listening Task were significantly positively correlated with AQ scores. The higher the AQ scores of individuals, the larger the amplitudes of this index of empathic neuronal habituation, suggesting that S2_P2_habituation was sensitive to the magnitude of autistic traits. In summary, these results indicate that S2_P2_habituation in the Passive Listening Task may be a suitable index to represent the empathic neuronal habituation to others’ vocal pain in individuals with autistic traits.

Altered habituation of empathic responses to others’ pain vocalisations was only found in the Passive Listening Task. This supports the first hypothesis that impaired empathic habituation can be found in individuals with autistic traits, if they have not been specifically instructed to focus their attention to others’ painful voices (regardless of whether pain or non-pain cues were presented). The effects of the modulation of top-down attention on emphatic responses were previously observed in individuals with ASD^29,30,37^. Without directly asking individuals who are involved in an explicitly empathic task, e.g., to give a verbal report (as applied in the Pain Judgment Task and Gender Judgment Task), the Passive Listening Task requires a more indirect assessment and implicit empathic processes^38^. The empathic competences of individuals with ASD and autistic traits have often been measured with regard to isolated and specific skillsets under explicit laboratory conditions^37^. For example, a study might focus on participants’ attention to emotional stimuli on a computer screen or to an audio recording, as used in the Pain Judgment Task and Gender Judgment Task in the present study. However, it remains highly debatable whether such an assessment can predict the participant’s spontaneous behaviour under natural conditions. A relevant study suggested that the cognitive assessment of others’ emotions by individuals with ASD and autistic traits remains basically intact^39^. Their impaired empathic affective sharing responses may be due to shortcomings of the automatic sensitivity to others’ emotions or their intrinsic motivation to perceive others’ feelings^40,41^. Similar empathic neuronal responses between High-AQ and Low-AQ groups were found in the Pain Judgment Task and the Gender Judgment Task. In the present study, the Passive Listening Task more likely reflects day-to-day life. Participants’ responses were not based on isolated explicit requests in structured situations, which is generally the result of empirical research^42^. Altered empathic neural habituation of High-AQ groups was only observed in the Passive Listening Task, which may explain the informants’ reports of spontaneous empathic behaviour in unstructured situations. This method is generally relied upon by diagnostic assessment procedures^37^.

In summary, the present study did not find global impairment of empathic habituation to others’ pain in individuals with autistic traits. In contrast, these individuals exhibited altered habituation to more intense, negative, and highly intense stimuli, i.e., painful voices in the present study, but not to less intense neutral voices. In addition, altered neural responses were found in the second and third repeated stimuli, but not in the first stimuli. This may be because the repetition of the painful voices is more intense than the single stimuli. Furthermore, this impairment was only observed when participants with autistic traits were passively listening. This finding may be because when the participants were not instructed to focus on the stimuli, the highly intense, repeated, and painful voices may become more disturbing. Thus, the pattern may be explained by the “Intense World Theory”^43^. This theory holds that autistic individuals may in general exhibit enhanced perception, attention, and memory capabilities, which may cause the world to become too intense and even aversive, causing many of the autistic symptoms, such as social interaction^44^ and perception^45^ disorders. Therefore, the social impairment of individuals with autistic traits may not be due to deficits in their ability to process others’ emotions and feelings. Rather, this social impairment is more likely the result of repeated social stimuli that are overly intense and excessively processed with altered habituation^43^.

Despite these potential implications, several limitations of the present study should also be noted. First, this study used tracks of three identical voices with constant and short ISI to evaluate participants’ empathic habituation. Further investigations should use dynamic videos to evaluate the individual empathic process, since these may be more similar to experiences of everyday life. Second, although the influence of autistic traits on the top-down attention modulation of empathic responses was assessed under experimental settings, whether and how these responses relate to real-world empathy requires further investigation. Third, in the Passive Listening Task, since filler trials were not integrated to assess whether participants focused sufficiently over the whole task, further investigations should evaluate the participants’ attentional process by integrating filler trials.

## Conclusions

In summary, this study investigated the influence of autistic traits on top-down attention-induced modulation with regard to the empathic neural habituation to others’ vocalization of pain. ERPs were used to measure the habituation that was induced by the repetition of three identical audio recordings (S1-S2-S3) in both High-AQ and Low-AQ groups. Compared with Low-AQ groups, the amplitudes of S2_P2_habituation were only significantly larger in High-AQ groups in the Passive Listening Task. This reflects the altered neural empathic habituation to others’ audio pain when High-AQ participants have not been instructed to focus their attention to others. These results suggest that individuals with autistic traits are affected by altered habituation of implicit neural empathic responses to others’ audible pain.

## Materials and methods

### Participants

A total of 2,083 university students at the Chongqing Normal University, aged 18–23 (mean = 19.30 years, SD = 1.14 years) were recruited to complete the Mandarin Version of the AQ questionnaire^11,46^. This questionnaire was used to estimate their autistic traits. Then, a subset of participants (those exhibiting the top 10% and bottom 10% of AQ scores)^16,17^ were randomly selected and divided into High-AQ (*n* = 20) and Low-AQ (*n* = 20) groups, respectively. The demographic characteristics of the participants in the High-AQ and Low-AQ groups are summarized in Table 2. None of the participants had been previously diagnosed with a medical, neurological or psychiatric disorder. At the time of the study, all participants had normal or corrected-to-normal vision and hearing. All participants signed informed consent forms after receiving a complete description of the study. In accordance with the Declaration of Helsinki, all participants gave their free and informed consent to participate in the study before the experiment, and all procedures were approved by the Chongqing Normal University research ethics committee. The procedures were performed in accordance with ethical guidelines and regulations.

**Table 2:** Psychometric variables for participants in both the High-AQ and Low-AQ groups.

	**High-AQ**	**Low-AQ**	**Statistics**
Gender (F/M)	10/10	10/10	
Age (years) (M ± SD)	19.40 ± 1.31	19.20 ± 0.95	*t*_*(38)*_ = − 0.551; *p* = 0.585
AQ Score (M ± SD)	29.50 ± 1.95	13.00 ± 1.82	*t*_*(38)*_ = − 21.539; *p* < 0.001

AQ = Autism Spectrum Quotient. Results (*t* values and *p* values) were obtained using independent sample *t*-tests between participants in the High-AQ and Low-AQ groups.

### Vocal stimuli

A total of 20 audio recordings of interjections (/ɑ/), spoken in either a painful (10 recordings) or neutral (10 recordings) prosody, were selected from the Montreal Affective Voices database. This database was recorded by 10 actors (five male and five female)^47^. All audio recordings were edited to last 700 ms, with a mean intensity of 70 dB^48^.

### Experimental procedure

The participants were seated in a quiet room with an ambient temperature of approximately 20 °C. In line with our previous studies^8,17^, top-down attention for another individual’s pain was manipulated by instructing the participants to either pay attention to pain cues or non-pain cues, or by passively listening to the provided audio recordings. They were instructed to participate in three experimental tasks: (1) a Pain Judgment Task, (2) a Gender Judgment Task, and (3) a Passive Listening Task. The order in which these three tasks were presented was counterbalanced across participants and the order of stimulus presentation was also randomized. Stimulus presentation was controlled using E-Prime (3.0) software.

**Figure 5:**
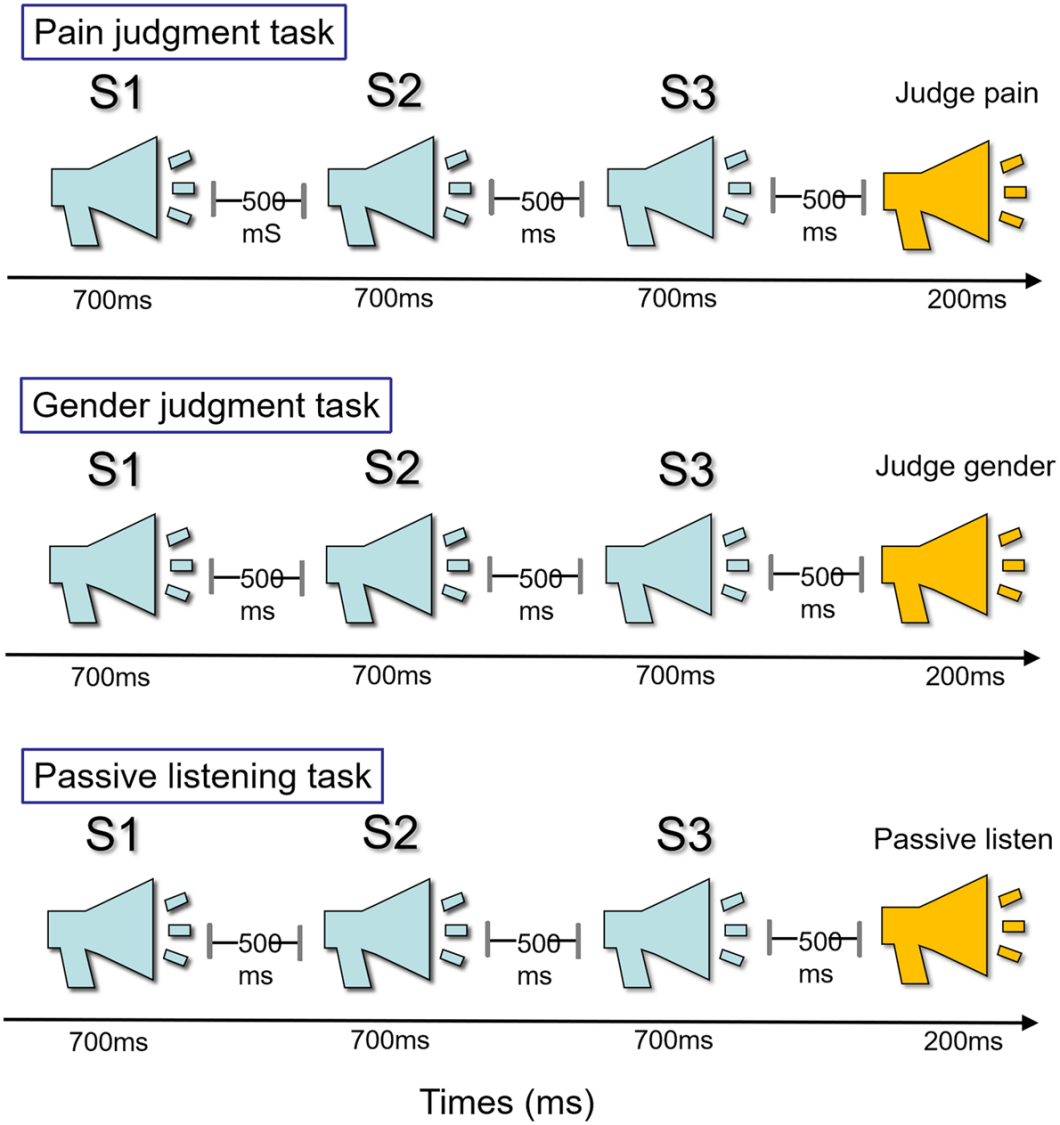
Schematic illustration for the experimental design. Top panel: Procedure of the Pain Judgment Task. Middle panel: Procedure of the Gender Judgment Task. Bottom panel: Procedure of the Passive Listening Task.

In the Pain Judgment Task (see the top panel in Fig. 5), participants were instructed to determine whether the recorded repeated voices sounded painful or neutral. Similar to previous studies^26,32,33^, each recorded section consisted of three identical stimuli (S1–S2–S3, as a triplet). Each voice in the triplet lasted for 700 ms, with an interval of 500 ms. Following the stimulus triplet, participants were instructed to respond as accurately and quickly as possible to a sound signal (“click”, 500 ms after the S3) by pressing a specific key (either “1” or “2”). The Pain Judgment Task consisted of two blocks, with 70 stimulus recordings (35 each for painful and neutral voices) in each block. The inter-trial interval was 3–4 s. Prior to the formal task, each participant took part in a training session to become familiarized with the process.

In the Gender Judgment Task (see the middle panel in Fig. 5), recordings of vocal stimuli (S1-S2-S3) were presented. Again, participants were instructed to press a key (“1” or “2”), as accurately and quickly as possible, to indicate whether the speaker was female or male. In the Passive Listening Task (see the bottom panel in Fig. 5), recordings of vocal stimuli (S1-S2-S3) were presented. This time, participants were instructed to passively listen to the audio recordings, but they were not required to make any response. Except for different task instructions, all experimental procedures (including stimulus categories, duration, and interval) were identical for these three tasks.

### EEG collection

In line with our previous study^17^, electroencephalography (EEG) data were recorded from 64 scalp sites using tin electrodes mounted on an elastic cap (Neuroscan4.3, Neurosoft, Inc., Sterling, VA, USA; passband: 0.01–100 Hz; sampling rate: 1,000 Hz). The electrode at the right mastoid was used as recording reference; that on the medial frontal aspect was used as ground electrode. Vertical electrooculograms (EOGs) were recorded both supra- and infraorbitally at the left eye. Horizontal EOGs were recorded as the left versus right orbital rim. All electrode impedances remained below 5 kΩ.

### EEG data analysis

The EEG data were pre-processed and analysed via MATLAB R2014a (MathWorks, USA) and the EEGLAB toolbox^49^. Continuous EEG signals were band-passed filtered (0.1–40 Hz) and segmented using a 4,100 ms time window relative to the onset of the first stimulus in the triplet (S1), with 500 ms pre-stimulus and 3,600 ms post-stimulus. All EEG epochs were baseline-corrected using the pre-stimulus interval. In addition, the EEG epochs were also visually inspected, and epochs contaminated by gross movements were removed. The removed EEG epochs constituted 7 ± 3.5% of the total number of epochs. EOG artefacts were corrected via the independent component analysis (ICA) algorithm^50^.

The EEG epochs elicited by painful and neutral voice recordings during the three experimental tasks were averaged and time-locked to the onset of the triplet of S1–S2–S3, yielding six averaged waveforms. Single-participant waveform averages were further averaged to obtain group-level waveforms, and group-level scalp topographies at corresponding peak latencies were computed by spline interpolation. Based on the topographical distribution of grand averaged ERP activity and previous studies^8,48^, the dominant ERP components involved in hearing others’ painful and neutral voices were identified, including N1, P2, and the late negative complex (LNC) elicited by each voice. Specifically, N1 and P2 waves were respectively defined as the most negative and positive deflections, respectively, at 100–300 ms after voice stimulus onset, with maximum distribution at the frontal-central electrodes. The LNC wave was the long-lasting negative and frontal-central distributed deflections within latency intervals of 400–700 ms after voice stimulus. As a result, for each participant and audio recording, N1 and P2 amplitudes were measured at the frontal-central electrodes (N1: Fz, F1, F2, FCz, FC1, FC2; P2: FCz, FC1, FC2, Cz, C1, C2) and calculated as the average ERP amplitudes within the latency interval ± 10 ms relative to the corresponding peak latency; LNC were measured at the frontal-central electrodes (Fz, F1, F2, FCz, FC1, FC2) and at latency intervals of 300–700 ms after the onset of the audio recording.

### Statistical analysis

Behavioural data, including ACCs and RTs, were compared. This was done using a three-way mixed design analysis of variance (ANOVA), with within-participant factors of “stimulation” (painful vs. neutral) and “task” (Pain Judgment Task, Gender Judgment Task), as well as the between-participants factor of “group” (High-AQ vs. Low-AQ).

Amplitudes of ERP components (N1, P2, and LNC) for S1–S2–S3 stimuli were compared via a three-way ANOVA with within-participant factors of “stimulation” (painful vs. neutral) and “task” (Pain Judgment Task, Gender Judgment Task, and Passive Listening Task), as well as the between-participants factor of “group” (High-AQ vs. Low-AQ). When a significant interaction effect was found, post hoc pairwise comparisons between High-AQ and Low-AQ groups were performed. The degrees of freedom for F-ratios were corrected according to the Greenhouse–Geisser method. To account for the multiple comparison problem, the *p* values were corrected using a false discovery rate (FDR) procedure^51^. In addition, to explore the effects of the modulation of top-down attention on the empathic responses between groups in response to painful voices, additional data analysis of the differences between Passive Listening Task and Pain Judgment Task in response to painful voices was conducted using Paired-Samples *t* Tests (for detailed statistical comparisons, see S. Table 2, Supplementary Materials).

To explore the empathic neural habituation effect to others’ vocal pain, indexes for empathic neural habituation were defined by computing the difference between the ERP amplitudes elicited by S2 or S3 and those elicited by S1 (S2_index_habituation and S3_index_habituation, respectively). For example, S2_index_habituation for the N1 component was calculated as S2_N1_habituation = S2_N1–S1_N1; S3_index_habituation for P2 component was calculated as S3_P2_habituation = S3_P2–S1_P2. For indexes of empathic neural habituation, the amplitudes of the differences between painful and neutral stimuli (painful – neutral) were compared via two-way ANOVA with the within-participant factors of “task” (Pain Judgment Task, Gender Judgment Task, and Passive Listening Task) and the between-participants factor of “group” (High-AQ vs. Low-AQ). In addition, to investigate the relationship between the indexes of empathic neural habituation that correlate with the AQ scores, the Pearson Correlation was calculated between them.

### Ethics approval and consent to participate

This research was approved by the Chongqing Normal University research ethics committee. All participants signed informed consent forms after receiving a complete description of the study. The ethics committee approved this consent procedure.

## Supplementary information

Supplementary information

## Data Availability

Supplementary data associated with this article can be found online, at https://pan.baidu.com/s/1_ne_a9mwsHUkcyTTeZXVpQ.
