# Supplementary material for: Altered neuronal habituation to hearing others’ pain in adults with autistic traits
Source: Sci Rep. 2020 Sep 14;10:15019. doi: 10.1038/s41598-020-72217-x (PMC7490706; doi:10.1038/s41598-020-72217-x)
Supplement: Supplementary file 1 — Supplementary information [file 41598_2020_72217_MOESM1_ESM.pdf]

Supplementary materials for "Altered Neuronal Habituation to Hearing Others' Pain in Adults with Autistic Traits"

Jing Meng[1,2], **Zuoshan Li**[1,2], **and Lin Shen**[3,*]

[1]Key Laboratory of Applied Psychology, Chongqing Normal University, Chongqing, China
[2]School of Education, Chongqing Normal University, Chongqing, China
[3]School of Mathematical Sciences, Chongqing Normal University, Chongqing, China

[*]corresponding author: linshen@cqnu.edu.cn

Behavioural data Statistical analysis

Data analyses were performed on both procedures. First, the recorded accuracies (ACCs) and reaction times (RTs) for audio recordings were compared via three-way repeated-measures analyses of variance (ANOVA), using two within-participant factors of "stimulation" (painful vs. neutral) and "task" (Pain Judgment Task vs. Gender Judgment Task), as well as the between-participants factor of "group" (High-AQ vs. Low-AQ). If the interaction effect was significant, post hoc two-way repeated measures ANOVA was performed for Low-AQ and High-AQ groups, with within-participants factors "task" and "stimulation". The data were analyzed using SPSS 15 software.

Behavioral results

Results of descriptive statistics of behavioral measures were showed in S. Table 1. ACCs and RTs were compared via three-way repeated-measures ANOVA, using the factors of "stimulation" (painful vs. neutral), "task" (Pain Judgment Task vs. Gender Judgment Task), and "group" (High-AQ vs. Low-AQ). ACCs were significantly modulated by the main effect of "stimulation" ($F_{1, 38}$ = 33.15, p < 0.001, η_p^2 = 0.47), ACCs were larger to the painful voices (87.8 ± 1.8 %) than the neutral voices (76.7 ± 1.6 %). ACCs were significantly modulated by the interaction of "stimulation × task" ($F_{1, 38}$ = 83.81, p < 0.001, η_p^2 = 0.69). Simple effects analyses indicated that, in the Pain Judgment Task, ACCs were significantly larger in response to the painful voices (95.7 ± 1.2 %) than neutral voices (67.0 ± 2.0 %) ($F_{1, 38}$ = 191.72, p < 0.001, η_p^2 = 0.84), whereas in the Gender Judgment Task, no difference were found between painful (80.0 ± 3.2 %) and neutral voices (86.5 ± 2.5 ms) ($F_{1, 38}$ = 4.09, p = 0.050, η_p^2 = 0.10).

RTs were significantly modulated by the main effect of "task" ($F_{1, 38}$ = 5.02, p = 0.040, η_p^2 = 0.11), RTs were longer in the Gender Judgment Task (688.62 ± 44.27 ms) than the Pain Judgment Task (626.04 ± 39.53 ms). RTs were significantly modulated by the interaction of "stimulation × task" ($F_{1, 38}$ = 8.81, p = 0.005, η_p^2 = 0.19). Simple effects analyses indicated that, in the Pain Judgment Task, RTs were significantly shorter in response to the painful voices (610.70 ± 39.64 ms) than neutral voices (641.38 ± 40.51 ms) ($F_{1, 38}$ = 5.45, p = 0.025, η_p^2 = 0.13), whereas in the Gender Judgment Task, no difference were found between painful (696.58 ± 45.80 ms) and neutral voices (680.66 ± 43.53 ms) ($F_{1, 38}$ = 1.71, p = 0.199, η_p^2 = 0.04).

S. Table 1. Results of descriptive statistics of behavioural measures.

Task	Group	ACC (%)		RT (ms)	
		Neutral voices	Painful voices	Neutral voices	Painful voices
Pain Judgment Task	Low-AQ	82.0 (21.2)	78.5 (22.1)	714.62 (301.63)	730.15 (302.28)
	High-AQ	91.1 (8.3)	81.4 (17.6)	646.70 (246.24)	663.02 (276.49)
Gender Judgment Task	Low-AQ	65.1 (10.7)	68.8 (14.4)	684.25 (311.70)	652.62 (291.72)
	High-AQ	96.2 (6.9)	95.3 (8.5)	598.51 (184.66)	568.77 (201.53)

Note. Mean response ACCs (%) and RTs (ms) (Mean (Standard deviation)) in Pain Judgment Task and Gender Judgment Task were present in the table.

S. **Table 2.** Summary of statistical analysis of amplitudes of painful voices between Passive Listening Task and Pain Judgment Task

	Passive Listening Task (Mean ± SD)	Pain Judgment Task (Mean ± SD)	$t_{(39)}$	*p*
S1_N1	-5.75 ± 3.69 μV	-5.76 ± 3.62 μV	0.02	0.988
S2_N1	-3.57 ± 6.06 μV	-5.17 ± 4.40 μV	1.75	0.087
S3_N1	-1.20 ± 5.62 μV	-1.37 ± 4.50 μV	0.19	0.854
**S1_P2**	**2.68 ± 3.02 μV**	**3.99 ± 3.51 μV**	**-3.24**	**0.002**
S2_P2	-0.88 ± 5.52 μV	-1.66 ± 4.00 μV	0.96	0.345
S3_P2	-2.07 ± 5.67 μV	-1.86 ± 4.56 μV	-0.22	0.825
S1_LNC	-5.03 ± 4.61 μV	-5.56 ± 3.97 μV	0.81	0.421
S2_LNC	-4.74 ± 5.98 μV	-5.38 ± 4.46 μV	0.73	0.469
S3_LNC	-4.37 ± 6.49 μV	-4.32 ± 4.99 μV	-0.04	0.967

Notes: statistics were obtained using Paired-Samples *t* Tests. The significant ($p < 0.05$) comparisons were shown in boldface.

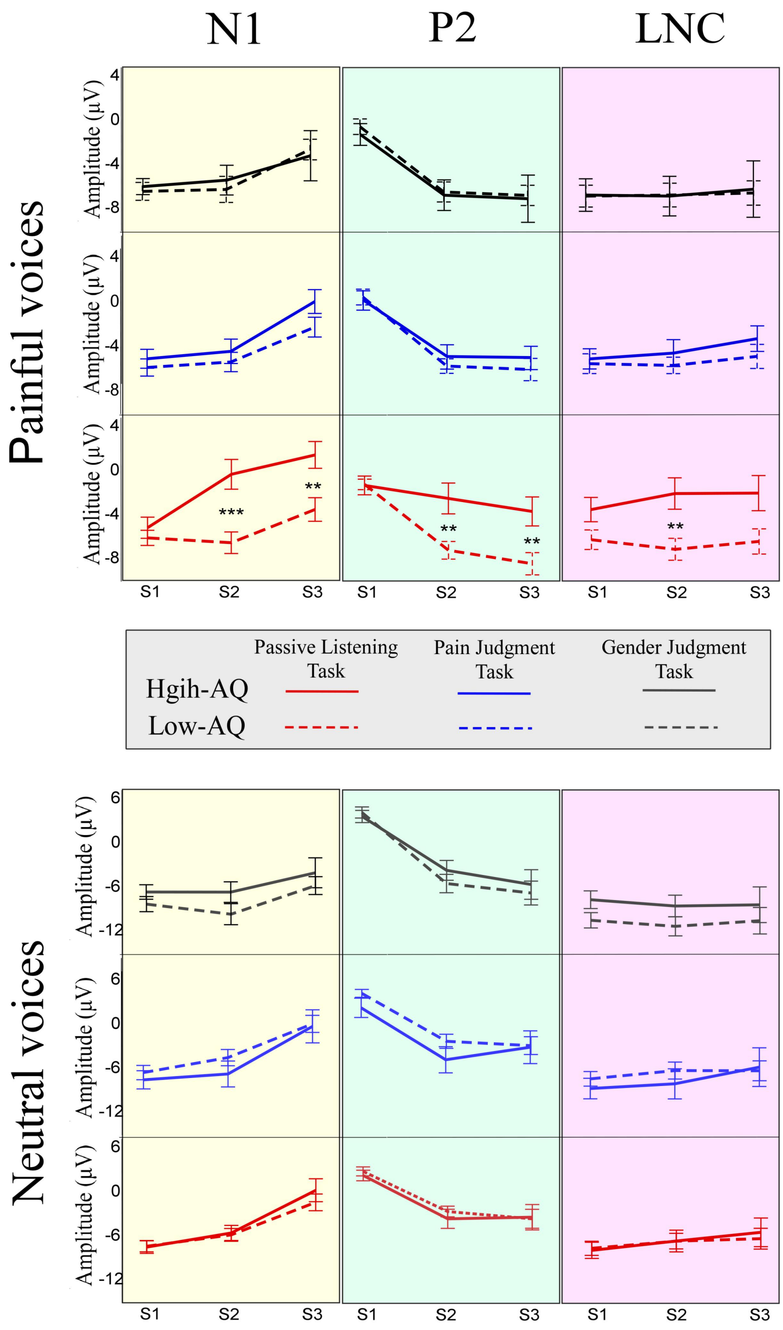

S. Figure 1: Comparisons of amplitudes of ERP components between groups

The amplitudes of N1 (left panel), P2 (middle panel), and LNC (right panel) elicited by the onset of S1, S2, and S3 audio recordings were compared between High-AQ (solid lines) and Low-AQ (dotted lines) groups during the Pain Judgment Task (blue lines), Gender Judgment Task (grey lines), and Passive Listening Task (red lines) in response to the painful (top panel) and neutral (bottom panel) voices. It is noticeable that High-AQ group exhibited altered neuronal habituation to the repeated painful voices during the Passive Listening Task. Data in the line charts were expressed as Mean ± SEM. **: $p < 0.01$, ***: $p < 0.001$.
